# Effect of i.p. insulin administration on IGF1 and IGFBP1 in type 1 diabetes

**DOI:** 10.1530/EC-13-0089

**Published:** 2014-01-23

**Authors:** P R van Dijk, S J J Logtenberg, K H Groenier, N Kleefstra, H J G Bilo, H J Arnqvist

**Affiliations:** 1 Diabetes Centre, Isala ClinicsPO Box 10400, Zwolle, 8000 G.K.The Netherlands; 2 Department of Internal MedicineUniversity Medical Center GroningenGroningenThe Netherlands; 3 Department of General PracticeUniversity Medical Center GroningenGroningenThe Netherlands; 4 Langerhans Medical Research GroupZwolleThe Netherlands; 5 Department of Internal MedicineIsala ClinicsZwolleThe Netherlands; 6 Division of Cell Biology, Department of Clinical and Experimental MedicineLinköping UniversityLinköpingSweden; 7 Faculty of Health SciencesDiabetes Research Centre, Linköping UniversityLinköpingSweden

**Keywords:** diabetes mellitus type 1, IGF1, IGFBP1, insulin, i.p. insulin infusion

## Abstract

In type 1 diabetes mellitus (T1DM), low concentrations of IGF1 and high concentrations of IGF-binding protein 1 (IGFBP1) have been reported. It has been suggested that these abnormalities in the GH–IGF1 axis are due to low insulin concentrations in the portal vein. We hypothesized that the i.p. route of insulin administration increases IGF1 concentrations when compared with the s.c. route of insulin administration. IGF1 and IGFBP1 concentrations in samples derived from an open-label, randomized cross-over trial comparing the effects of s.c. and i.p. insulin delivery on glycaemia were determined. T1DM patients were randomized to receive either 6 months of continuous i.p. insulin infusion (CIPII) through an implantable pump (MIP 2007C, Medtronic) followed by 6 months of s.c. insulin infusion or *vice versa* with a washout phase in between. Data from 16 patients who had complete measurements during both treatment phases were analysed. The change in IGF1 concentrations during CIPII treatment was 10.4 μg/l (95% CI −0.94, 21.7 μg/l; *P*=0.06) and during s.c. insulin treatment was −2.2 μg/l (95% CI −13.5, 9.2 μg/l; *P*=0.69). When taking the effect of treatment order into account, the estimated change in IGF1 concentrations was found to be 12.6 μg/l (95% CI −3.1, 28.5 μg/l; *P*=0.11) with CIPII treatment compared with that with s.c. insulin treatment. IGFBP1 concentrations decreased to −100.7 μg/l (95% CI −143.0, −58.3 μg/l; *P*<0.01) with CIPII treatment. During CIPII treatment, parts of the GH–IGF1 axis changed compared with that observed during s.c. insulin treatment. This supports the hypothesis that the i.p. route of insulin administration is of importance in the IGF1 system.

## Introduction

Insulin and insulin-like growth factor 1 (IGF1) are structurally and functionally closely related peptides. IGF1, mainly synthesized in the liver after stimulation of the GH receptor, plays a central role in cell metabolism and growth regulation (1, 2, 3). In plasma, IGF1 is bound to IGF-binding proteins (IGFBPs), among which IGFBP3 binds to ∼80% of the total amount of IGF1 present in the circulation. It is only the free fraction of IGF1, comprising <1% of the circulating IGF1, that is biologically active. IGFBP1 is produced in the liver and regulated acutely (in an inverse direction) by insulin, thereby allowing insulin to regulate IGF1 bioactivity (4, 5, 6, 7).

Through an up-regulation of hepatic GH receptor expression, insulin increases the hepatic sensitivity of GH stimulation and subsequently increases IGF1 production (8). Furthermore, insulin increases IGF1 bioactivity by a down-regulation of IGFBP1 expression in the liver (5). In type 1 diabetes mellitus (T1DM), with insufficient insulinization of the liver due to lack of endogenous insulin in the portal vein, there appears to be a dysfunction of the GH–IGF1 axis. This is characterized by low concentrations of total IGF1 and IGFBP3 and high concentrations of IGFBP1 and GH (9, 10, 11, 12, 13, 14). Although these abnormalities have been described in a situation of poor glycaemic control, exogenous s.c. insulin only attenuates these disturbances but does not completely reverse them (15, 16, 17, 18).

With continuous i.p. insulin infusion (CIPII), insulin is infused directly into the i.p. space and is almost entirely absorbed in the portal system, resulting in higher portal insulin concentrations, higher hepatic uptake and lower peripheral plasma insulin concentrations compared with those observed upon s.c. insulin administration (19, 20). This results in a more physiological mode of insulin administration compared with s.c. insulin administration and could thus have a beneficial effect on the impaired GH–IGF1 axis (21). In this study, we tested the hypothesis that intraperitoneally administered insulin when compared with s.c. insulin results in an increase in IGF1 concentrations in samples derived from a randomized cross-over trial (Supplementary Figures 1, 2, and 3, see section on supplementary data given at the end of this article).

## Subjects and methods

### Study design and population

The full study design has been published previously (22). In brief, the study from which the samples were derived had an open-label randomized, cross-over design and was conducted at a single centre (Isala Clinics, Zwolle, The Netherlands). The study consisted of four phases: the qualification phase, the first treatment phase, the cross-over phase and the second treatment phase. During a 3-month qualification phase, the patients' prestudy insulin therapy was used to attempt optimization of their glycaemic control. Patients with T1DM (aged 18–70 years with fasting C-peptide concentrations <0.20 nmol/l, HbA1c concentrations ≥58 mmol/mol and/or ≥5 incidents of hypoglycaemia (<4.0 mmol/l) per week and treated with multiple daily injections (MDIs) or continuous s.c. insulin infusion (CSII)) were randomly allocated to continue their current s.c. mode of therapy or start with i.p. insulin administration using an implantable pump. These two groups (start i.p. or continue s.c.) differed only in the sequence of the mode of insulin administration. Randomization was carried out using sealed non-transparent envelopes, with adequate blinding of the content of the envelopes. The patients were assigned to the treatment order as defined by the code in the envelopes (start i.p. or continue s.c.). The randomization system used blocks of 4. In the original study, 50 patients were screened for eligibility, of which 25 entered the qualification phase. In one patient, acceptable glycaemic control was reached during the qualification phase and thus 24 patients were randomly assigned to the first treatment phase; 12 patients were assigned to continue s.c. insulin and 12 patients to start with CIPII during the first phase of the trial. One patient, with CIPII at start, withdrew consent during the trial. In the present analysis, we included only patients with complete IGF1 results in both treatment phases; therefore, seven patients were excluded.

Insulin (U400 semi-synthetic human insulin of porcine origin; Hoechst, Frankfurt, Germany, nowadays Sanofi-Aventis) was administered with an implantable pump (MIP 2007C; Medtronic/Minimed, Northridge, CA, USA). The CIPII pump was implanted under general anaesthesia at the start of the CIPII phase in all the subjects. For subjects who received s.c. insulin during the second treatment phase, the CIPII pump was filled with an inert fluid at the end of the first treatment phase. S.c. insulin was delivered with either MDIs or CSII, according to what was used before the study.

Patients treated with MDIs continued to follow their own insulin regimen, i.e. rapid-acting insulin analogues before meals and a daily dose of long-acting insulin. Between both treatment phases of 6 months, a cross-over phase of 4 weeks was instituted to minimize the carry-over effects of CIPII treatment. During the cross-over phase, insulin was administered subcutaneously.

If the subject was using more than 40 IU of s.c. insulin per day before starting the CIPII phase of the study, his or her starting dose was set at 90% of the prior s.c. dose. Subjects using <40 IU of s.c. insulin received a starting dose of 80% of the prior s.c. dose. Initially, the dose was equally divided between a basal rate (50%) and a bolus before meals. During all study visits, the seven-point glucose readings were used to adjust the dose regimen if necessary to achieve preprandial glucose levels between 4.0 and 7.0 mmol/l and postprandial levels between 4.0 and 9.0 mmol/l. The patients were instructed not to start a specific diet or weight reduction programme during the trial.

### Measurements of clinical and biochemical parameters

Measurements were carried out at baseline, the end of the qualification phase, and at the start, at the halfway point, and at the end of both treatment phases. HbA1c concentrations were measured using a Primus Ultra2 using HPLC (reference value 20–42 mmol/mol). IGF1 and IGFBP1 concentrations, reported as μg/l, were measured in 1.5 cc serum samples collected at random and non-fasting at the start and end of each treatment phase and stored at −80 °C until analysis in 2011, carried out at the Department of Clinical and Experimental Medicine of the Linköping University, Linköping, Sweden. Total IGF1 concentration was measured using a one-step ELISA after acid–ethanol extraction from its binding protein using a commercial kit (Human IGFI Quantikine ELISA Kit R&D Systems) (23). Inter-assay coefficients of variation (CV) values were 10.9, 5.9 and 18.2% for high (278 μg/l), medium (116 μg/l) and low (45 μg/l) controls respectively. IGFBP1 concentration was measured with ELISA (human IGFBP1 DuoSet, DY871, R&D Systems, Minneapolis, MN, USA). The assay was carried out according to the protocol provided by the manufacturer. Microtiter plates, MaxiSorp (Nunc, Roskilde, Denmark), normal goat serum (Fisher Scientific) and tetramethylbenzidine dihydrochloride (Sigma Life Science) were used. The microtiter plates were coated overnight with capture antibody. Inter-assay CV values for high (1688 μg/l) and low (4 μg/l) controls were 7.8 and 20.0% respectively.

### Primary and secondary outcomes

The primary outcome of this *post hoc* analysis was the difference in IGF1 concentrations between the two treatment phases. Secondary outcomes were changes in IGFBP1 concentrations during both treatment phases, changes in IGF1 and IGFBP1 concentrations in patients with and without detectable C-peptide and correlations of changes in HbA1c concentrations, total insulin dose, C-peptide concentrations with IGF1 and IGFBP1 concentrations.

### Statistical analyses

To calculate the mean difference with a 95% CI, the Hills–Armitage approach was used, which accounts for any period effect. Linear mixed models (PROC MIXED, SAS 9.2; SAS Institute Inc, Cary, NC, USA) were used to test differences, taking treatment order into account. The assumption of normal distribution of the residuals was examined using *Q*–*Q* plots. In addition, *Q*–*Q* plots were used to determine whether the tested variable had a normal distribution. Correlations were investigated using the Pearson product–moment correlation coefficient or, when appropriate, non-parametric Spearman's *ρ*. Comparisons between outcomes during both treatment modalities were made using *t*-test for paired comparisons for IGF1 and Wilcoxon match-pair signed-rank tests for IGFBP1. Patients with and without detectable C-peptide were compared with unpaired *t*-test. IGFBP1 concentrations had a skewed distribution (right tail), and they are presented as median and interquartile range (IQR). Differences in IGFBP1 concentrations were normally distributed. Besides the linear mixed models, all the analyses were carried out using SPSS version 18.0, Inc. A (two-sided) *P* value of <0.05 was considered to be statistically significant.

### Ethical considerations

The study was carried out in accordance with the Declaration of Helsinki. Informed consent was obtained from all the patients for the initial study. The protocol was approved by the Medical Ethics Committee of the Isala Clinics in Zwolle. Additional informed consent was obtained for the present study.

## Results

### Patients

The study sample consisted of 16 patients, six males and ten females, with a median (IQR) age of 42.4 (30.4–49.4) years and diabetes for a duration of 21.7 (10.4–30.5) years. Three patients used MDIs and 13 CSII before the study, the qualification phase and the s.c. treatment phase. Mean IGF1 (±s.d.) concentrations at the start of the s.c. and i.p. insulin treatment phases were respectively 83.7±31.9 and 76.3±24.5 μg/l.

### IGF1 and IGFBP1 concentrations

**Figure 1:**
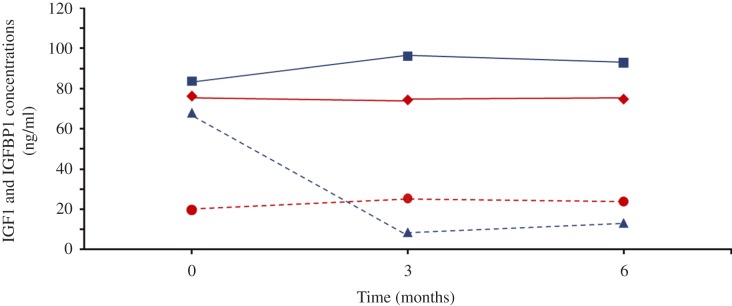
Course of mean IGF1 (consecutive line) and median IGFBP1 (dashed line) concentrations during 6 months of s.c. (red line) or i.p. (blue line) insulin treatment.

Results obtained for the IGF1 and IGFBP1 measurements during the different treatment modalities are summarized in Table 1 and Fig. 1. The observed IGF1 and IGFBP1 concentrations were significantly different between both treatment modalities at 3 and 6 months. No significant carry-over effects were observed for IGF1 (*P*=0.33) and IGFBP1 (*P*=0.83) concentrations between both treatment phases.

The estimated mean change in IGF1 concentrations during the CIPII phase was 10.4 μg/l (95% CI −0.94, 21.7 μg/l; *P*=0.06) and during the s.c. treatment phase was −2.2 μg/l (95% CI −13.5, 9.2 μg/l; *P*=0.69). When taking the effect of treatment order into account, the estimated difference in concentrations between the i.p. treatment phase and the s.c. treatment phase was found to be 12.6 μg/l (95% CI −3.1, 28.5 μg/l; *P*=0.11).

IGFBP1 concentrations decreased significantly during the i.p. treatment phase, −100.7 μg/l (95% CI −143.0, −58.3 μg/l; *P*<0.01), but not during the s.c. treatment phase, 9.4 μg/l (95% CI −33.0, 51.8 μg/l; *P*=0.64). The estimated difference between both phases was −110.4 μg/l (95% CI −170.0, −50.1 μg/l; *P*<0.01).

### Glycaemic control

HbA1c concentrations decreased with CIPII treatment from 68±16.5 to 60±6.6 mmol/mol after 3 months and remained stable at 6 months (61±9.9 mmol/mol). During the s.c. treatment phase, there was no change in glycaemic control. No significant carry-over effects were observed between both treatment phases (*P*=0.05). HbA1c concentrations improved to −10.0 mmol/mol (95% CI −18.4, −1.6; *P*=0.02) with CIPII treatment than with s.c. insulin treatment. During the i.p. treatment phase, changes in HbA1c concentrations correlated with changes in IGF1 concentrations (*r*=−0.5, *P*=0.04), but not with those in IGFBP1 concentrations (*r*=−0.3, *P*=0.33).

### Total insulin dose, C-peptide concentrations and associations with IGF1 and IGFPBP1 concentrations

Mean daily insulin dose decreased with i.p. treatment with −2.0 IU/day (95% CI −13.7, 9.6 IU/day; *P*=0.71) compared with s.c. insulin treatment. Spearman's correlation coefficient indicated a non-significant association between the mean difference in insulin dose and IGF1 concentrations during the i.p. treatment phase (*r*=−0.02, *P*=0.95). Changes in IGFBP1 concentrations did not correlate with changes in total insulin dose (*r*=0.19, *P*=0.48) during the i.p. treatment phase. Changes in IGF1 and IGFBP1 concentrations during the CIPII phase did not exhibit any significant correlation (*r*=−0.23, *P*=0.40).

There was no significant difference in the change in IGF1 concentrations during the i.p. treatment phase between patients with undetectable (≤0.01 nmol/l, *n*=6) and detectable (>0.01 nmol/l, *n*=10) C-peptide: 12.6±22.2 vs 3.7±22.1 ng/ml (*P*=0.45). IGFBP1 concentrations were −49.5 (−222.9, −17.4) and −57.7 (−182.7, −12.3) μg/l respectively. The association between the concentrations of C-peptide and the change in IGF1 concentrations during the i.p. or s.c. treatment phase was also not significant: *r*=−0.02 (*P*=0.94) and *r*=−0.16 (*P*=0.56).

## Discussion

Concentrations of IGFBP1 decreased significantly during CIPII treatment compared to s.c. treatment. IGF1 concentrations did not change significantly during the i.p. treatment phase, and this change was also not significant when compared with that observed during intensive s.c. insulin treatment.

As there is (almost) no insulin production in patients with T1DM, it has been hypothesized that low insulin concentrations in the portal vein cause decreased IGF1 concentrations/bioactivity through both GH receptor- and IGFBP1-mediated mechanisms (5, 8). In all three studies of the IGF system in which subjects with T1DM were treated with i.p. insulin infusion, an increase in IGF1 concentrations was observed. Shishko *et al*. reported the normalization of plasma IGF1 concentrations with intraportal infusion of insulin in newly diagnosed T1DM patients (18). Unfortunately, that study lacked data regarding the presence or absence of endogenous production of insulin. A longitudinal study carried out by Hanaire-Broutin *et al*. (18) demonstrated a steady increase in plasma IGF1 concentrations to a low-normal level 1 year after the initiation of CIPII treatment, despite a lack of improvement in HbA1c concentrations. In the present study, IGF1 concentrations were significantly higher after 3 and 6 months with CIPII treatment compared with those observed with s.c. insulin treatment and a non-significant change of 10.4 μg/l was observed within the i.p. treatment period of 6 months. Compared with that observed during s.c. insulin treatment, this change was not significant. These findings may be due to the size of the sample (*n*=16) and/or the duration of the present study. In the study carried out by Hanaire-Broutin, IGF1 concentrations tended to increase even after 6 months. In severely uncontrolled diabetes, IGF1 concentrations are low (24), but ordinary glycaemic control probably has little effect on IGF1 concentrations, as suggested by this study and shown by Hedman *et al*. (17) earlier.

At the start of the CIPII treatment phase, several patients had very high IGFBP1 values. Due to these outliers, the IGFBP1 concentrations at the start of the i.p. treatment phase were high. Of interest, all five patients with IGFBP1 concentrations >150 μg/l (range: 181.2–330.0 μg/l) were in the ‘i.p. first’ cross-over group. It was remarkable that additional analysis indicated a significantly longer median duration between pump implantation and IGFBP1 measurement for these five patients compared with the other patients (0.5 vs 0.0 years; *P*<0.001). Therefore, we hypothesize that the high IGFBP1 concentrations in these five individuals represent an acute effect in the start-up phase of i.p. insulin. It has been reported that insulin withdrawal for 8 h in patients with type 1 diabetes treated with CSII increased IGFBP1 concentrations sixfold, and it is conceivable that the high IGFBP1 values could be due to a lag in insulin delivery (4). Nevertheless, *post hoc* analysis of patients with IGFBP1 concentrations <150 μg/l still indicated that the change in IGFBP1 concentrations during the i.p. treatment phase remained significant (−46.3 μg/l, 95% CI −80.2, −12.4 μg/l; *P*=0.01) and, as a right skew could influence the estimated difference between the treatment modalities, that the estimated difference between the treatment groups still remained at −49.9 μg/l (95% CI −97.9, −1.92 μg/l; *P*=0.04). When paired comparisons of IGFBP1 concentrations were made during treatment at 3 and 6 months, IGFBP1 concentrations were found to be lower with CIPII treatment than with CSII treatment. The lowering of IGFBP1 concentrations suggests an increase in free IGF1 concentrations, i.e. IGF1 bioactivity by CIPII treatment (1). As there was no increase in insulin dose, this is compatible with an enhanced insulin effect on the liver by CIPII treatment (3). The observed decrease in IGFBP1 concentrations in the present study is in line with previous reports and, as IGFBP1 concentrations correlate with GH secretion and hepatic glucose production, may indicate the importance of the i.p. route of insulin administration (25, 26, 27).

For the interpretation of the results of this study, it must be acknowledged that the original study was powered to detect differences in hypoglycaemic events between i.p. and s.c. insulin and not in IGF1 or IGFBP1 concentrations. In contrast to the studies carried out by Shishko and Hanaire-Broutin (18, 25), samples were collected randomly and information about the antecedent insulin dose was lacking . Finally, lack of a large reference population impairs the comparison of the IGF1 concentrations found in the present study with those of healthy subjects.

Although the clinical significance of low IGF1 concentrations in patients with T1DM remains unclear at present, CIPII could have an additional benefit on top of glycaemic control by altering the dysregulated GH–IGF system by increasing portal insulin concentrations. This is a hypothesis worth testing in future research.

## Supplementary data

This is linked to the online version of the paper at http://dx.doi.org/10.1530/EC-13-0089.

## Author contributions

P v D was responsible for study design and drafting and reviewing the manuscript and carried out the statistical analyses; S L was responsible for study design, inclusion, and drafting and reviewing the manuscript; N K and H B were responsible for study design and reviewed the manuscript, K G carried out the statistical analyses and reviewed the manuscript; and H A was responsible for drafting and reviewing the manuscript.

## Figures and Tables

**Table 1:** Observed IGF1, IGFBP1 and HbA1c concentrations and estimated changes during s.c. and i.p. insulin treatment. IGF1 and HbA1c concentrations are presented as mean (s.d.) and IGFBP1 concentrations are presented as median (IQR). *n*=16 for IGF1, IGFBP1 and HbA1c at all time points.

	**IGF1** (μg/l)		**IGFBP1** (μg/l)		**HbA1c** (mmol/mol)	
CIPII	SC	CIPII	SC	CIPII	SC
0 months^a^	83.7 (31.9)	76.3 (24.5)	68.0 (35.3, 213.6)	19.7 (11.9, 52.0)*	68 (16.5)	68 (15.4)
3 months	96.1 (44.9)	74.4 (28.0)*	8.5 (5.8, 14.4)	25.5 (9.5, 45.5)*	60 (6.6)	70 (14.3)*
6 months	92.9 (39.3)	74.8 (16.0)*	13.2 (6.6, 22.1)	23.9 (14.6, 56.2)*	61 (9.9)	69 (18.7)*
Change^b^	10.4 (−0.94, 21.7; *P*=0.06)	−2.2 (−13.5, 9.2; *P*=0.69)	−100.7 (−143.0, −58.3; *P*<0.01)	9.4 (−33.0, 51.78; *P*=0.64)	−8.9 (−14.4, −3.3; *P*<0.05)	0.8 (−5.6, 6.7; *P*=0.78)

*P<0.05 for CIPII vs SC at that time point.

a 0 months: at the end of the 3-month qualification phase.

b Estimated mean changes in IGF1, IGFBP1 (both in μg/l) and HbA1c (mmol/mol) concentrations per treatment modality (95% CI).
